# Differentiation of glioblastoma tissues using spontaneous Raman scattering with dimensionality reduction and data classification

**DOI:** 10.3389/fonc.2022.944210

**Published:** 2022-09-15

**Authors:** Igor Romanishkin, Tatiana Savelieva, Alexandra Kosyrkova, Vladimir Okhlopkov, Svetlana Shugai, Arseniy Orlov, Alexander Kravchuk, Sergey Goryaynov, Denis Golbin, Galina Pavlova, Igor Pronin, Victor Loschenov

**Affiliations:** ^1^ Prokhorov General Physics Institute of the Russian Academy of Sciences, Moscow, Russia; ^2^ National Research Nuclear University MEPhI (Moscow Engineering Physics Institute), Moscow, Russia; ^3^ N.N. Burdenko National Medical Research Center of Neurosurgery, Moscow, Russia; ^4^ Institute of Higher Nervous Activity and Neurophysiology of the Russian Academy of Sciences, Moscow, Russia

**Keywords:** glioblastoma multiforme, raman spectroscopy, dimensionality reduction, principal component analysis, biochemical components, optical biopsy

## Abstract

The neurosurgery of intracranial tumors is often complicated by the difficulty of distinguishing tumor center, infiltration area, and normal tissue. The current standard for intraoperative navigation is fluorescent diagnostics with a fluorescent agent. This approach can be further enhanced by measuring the Raman spectrum of the tissue, which would provide additional information on its composition even in the absence of fluorescence. However, for the Raman spectra to be immediately helpful for a neurosurgeon, they must be additionally processed. In this work, we analyzed the Raman spectra of human brain glioblastoma multiforme tissue samples obtained during the surgery and investigated several approaches to dimensionality reduction and data classificatin to distinguish different types of tissues. In our study two approaches to Raman spectra dimensionality reduction were approbated and as a result we formulated new technique combining both of them: feature filtering based on the selection of those shifts which correspond to the biochemical components providing the statistically significant differences between groups of examined tissues (center of glioblastoma multiforme, tissues from infiltration area and normally appeared white matter) and principal component analysis. We applied the support vector machine to classify tissues after dimensionality reduction of registered Raman spectra. The accuracy of the classification of malignant tissues (tumor edge and center) and normal ones using the principal component analysis alone was 83% with sensitivity of 96% and specificity of 44%. With a combined technique of dimensionality reduction we obtained 83% accuracy with 77% sensitivity and 92% specificity of tumor tissues classification.

## 1 Introduction

Brain tumors such as gliomas, and especially glioblastomas, are the hardest ones to determine the borders and surgically remove. Precise identification and total disposal of brain tumors are significant for radical removal while preserving the surrounding healthy tissue as much as possible. Studies, including our own, have shown that the content of 5-ALA-induced protoporphyrin IX (Pp IX) in glial brain tumors is a highly specific criterion for demarcating tumor boundaries, as well as determining the degree of its malignancy (1). However, in 30% of cases, we observed only a slight accumulation of Pp IX in tumor cells, only 10–20% of low-grade gliomas showed visible fluorescence upon administration of 5-ALA, which does not allow using it as an exhaustive criterion for determining tumor tissues. In this case, to demarcate the boundaries of low-grade gliomas, one can take the path of increasing the sensitivity of fluorescence analysis (2) or choosing another optical-spectral approach. For example, Raman spectroscopy is a sensitive and fast tool for analyzing the molecular composition of tissues in order to differentiate between tumor and normal tissues.

Raman spectroscopy has become a powerful tool due to its ability to investigate biological samples with high molecular specificity without significant sample preparation. It is widely used to identify various biomolecules, including nucleic acids, proteins, carbohydrates, and lipids, both *in vitro* and *in vivo*. The fast advances in recent decades in laser and fiber optic technologies, as well as in chemometrics and machine learning that help extract information hidden from direct perception, have led to translation of the technologies of Raman spectroscopy into clinical conditions (3). The problem of increasing the radicality of intracranial glial tumor resection has led to the use of this method in neurosurgery. To date, various modes of Raman spectroscopy and tools that implement them have been used, including handheld probes for obtaining spontaneous Raman spectra *in vivo* during neurosurgery (4), spontaneous and stimulated Raman scattering microscopy of fresh human brain tumor specimens ex vivo (5, 6), resonance Raman spectroscopy for optical biopsy identification and grading of gliomas (7) and many other approaches (8). Resonant Raman scattering is observed when using exciting radiation, the frequency of which corresponds to a real, rather than a virtual, electronic transition. This leads to a significant increase in the signal, but is accompanied by a significant fluorescent background, which can be an obstacle for intraoperative navigation due to the presence of both endogenous and exogenous fluorophores in tissues. The use of stimulated Raman scattering limits us to the choice of the wavelength of the wavelength of Stokes component. Surface-enhanced methods also provide a significant increase in sensitivity, but signal amplification is achieved only in a thin layer near the substrate, which provides plasmon resonance, which limits the depth of tissue probing. Spontaneous Raman scattering is inferior in sensitivity to enhanced methods of Raman spectroscopy, however, the simplicity of signal detection makes it the most accessible for clinical applications.

Despite the advantages of Raman spectroscopy, it must be taken into account that the Raman spectra of biological systems are complex and diverse due to their heterogeneous nature, complex molecular composition and structure. Hence, the interpretation of the results obtained by Raman spectroscopy is difficult, and in order to overcome these difficulties and for a deeper understanding, we need to use various methods of data mining. The article (9) considers a large number of methods for preprocessing and postprocessing of Raman spectra. Among them the methods of dimensionality reduction play a crucial role (10). Sometimes minor differences can contain important information, but many peaks are common for both healthy and tumor tissues, that is, they do not contribute to their differentiation. In statistics, machine learning, and information theory, dimensionality reduction is the transformation of data that reduces the number of variables by deriving the principal variables. The transformation can be divided into feature selection and feature projection methods. The feature selection method tries to find a subset of the original variables (called features or attributes) (11). Feature projection transforms data from a high-dimensional space to a low-dimensional space. Data transformation can be linear, as in principal component analysis (PCA) (12), but there are many techniques for nonlinear dimensionality reduction. Non-linear approaches could be more powerful than PCA but they can be slow to optimize and they get different, locally optimal solutions each time while repeatability is important for clinical applications. Feature selection can be implemented through wrapping approaches, filtering algorithms, and nesting methods.

Filters usually require less complex computations than wrappers, but they give opportunity to use features that are not configured to a specific type of predictive model. To evaluate subsets of features, filter techniques appeal to a proxy metric instead of an error metric. The metric is chosen for easy computation without affecting the utility metric of the feature set. Usually applied are such metrics as correlation coefficient of mixed Pearson moments, mutual information, pointwise mutual information, the result of significance tests for each class/attribute combination or distance between classes/within a class.

In this work on the first step we have implemented a filter approach with the result of significance test as measure of feature extraction quality. As the classes under consideration, spectra were taken from samples from the center of the tumor, from the edge of the tumor (from the tumor cells infiltration area) and from the normally appeared white matter. The purpose of this consideration was to detect spectral features characteristic of tumor tissues and tissues at the tumor border.

Another less commonly used but powerful method is biochemical component analysis (BCA). In BCA, the Raman spectrum is considered by the contribution of several known biochemical components, such as proteins, lipids, nucleic acids, glycogen groups, etc. In work (13), the authors used both methods (PCA and BCA) to analyze the Raman spectra measured on living cells, apoptotic and necrotic leukemia cells. The comparison shows that the two methods give comparable sample classification accuracy when the number of principal components is the same. Changes in the contribution of biochemical components to BCA can be interpreted using the principles of cell biology during apoptosis and necrosis. On the contrary, the contribution of most of the principle components to the PCA is difficult to interpret, except for the first one. The ability of BCA to detect small biochemical changes in the spectra of cells and the excellent classification accuracy may prompt the widespread use of Raman spectroscopy in biological research.

In this work, we also focused on comparing and, moreover, on the combination of these two approaches, namely, comparing such groups of Raman spectroscopy data dimensionality reduction methods as feature projection methods (which include PCA) and feature filtering methods (which are used by BCA, when we select significant peaks corresponding to certain biochemical components).

## 2 Materials and methods

### 2.1 Studied tissue samples

The studied material was obtained during routine surgical removal of intracranial tumors (8 patients diagnosed with glioblastoma multiforme, 10 samples in total) at N.N. Burdenko National Medical Research Center of Neurosurgery. The samples were kept in saline at 4°C in a sealed tube for no more than three hours after removal. Before the Raman investigation, the material was removed from the tube and placed on a sterilized aluminum foil to remove the potential Raman spectrum of the substrate, where it was observed with the Raman probe. After this, the sample was put into an Eppendorf tube containing formalin and sent for histological investigation. As a result, the samples were separated into three groups: normal brain tissue (3 samples), tumor edge (3 samples), tumor center (4 samples). The localization was reported by the operating surgeon.

### 2.2 Set-up for registration and processing of spontaneous Raman spectra

**Figure 1:**
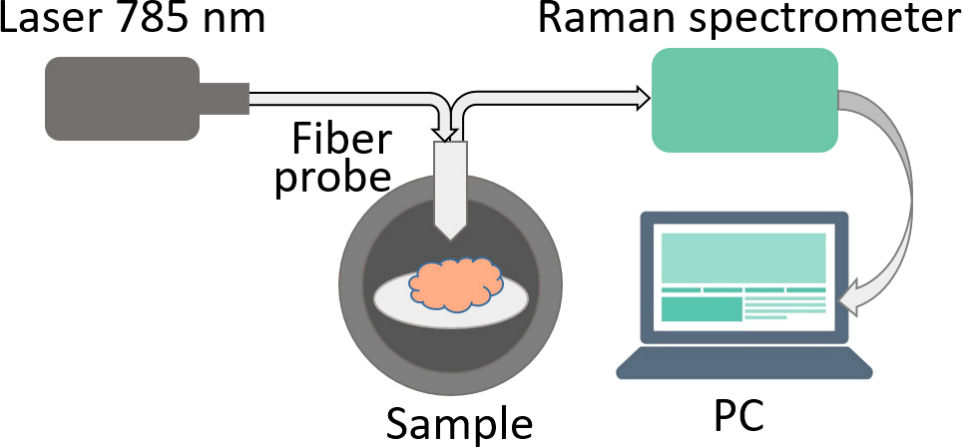
Raman spectra measurement setup.

Raman spectra were measured using a Raman spectrometer Raman-HR-TEC-785 (StellarNet, USA) under excitation with 785 nm laser radiation from Ramulaser™-785 laser source (StellarNet, USA) delivered with a fiber-optic confocal probe (**Figure 1**).

All measurements were conducted in a darkened room. Prior to measuring the Raman signal from each sample, the background signal was measured 5 times with 30 sec exposition, then averaged. Each sample was measured 5 times at various angles. Spectra where the measured signal exceeded 90% of the spectrometer dynamic range were excluded to avoid errors due to oversaturation. As a result, the total number of measurements was 14 for normal brain tissue, 15 for tumor edge and 19 for tumor center.

Each measured spectrum consisted of 2050 points in the range of 127–2830 cm^-1^. For each sample, the previously measured background spectra were averaged. This average background spectrum was subtracted from each Raman spectrum to remove the background illumination. Each spectrum was then smoothed to reduce noise using a Savitzky-Golay filter (15 pixel width, 3rd order polynomial) from SavitzkyGolay.jl Julia language package. The resulting spectra were characterized by intense fluorescence signal, which was approximated as a sum of Morlet wavelets (14) and removed (15).

**Figure 2:**
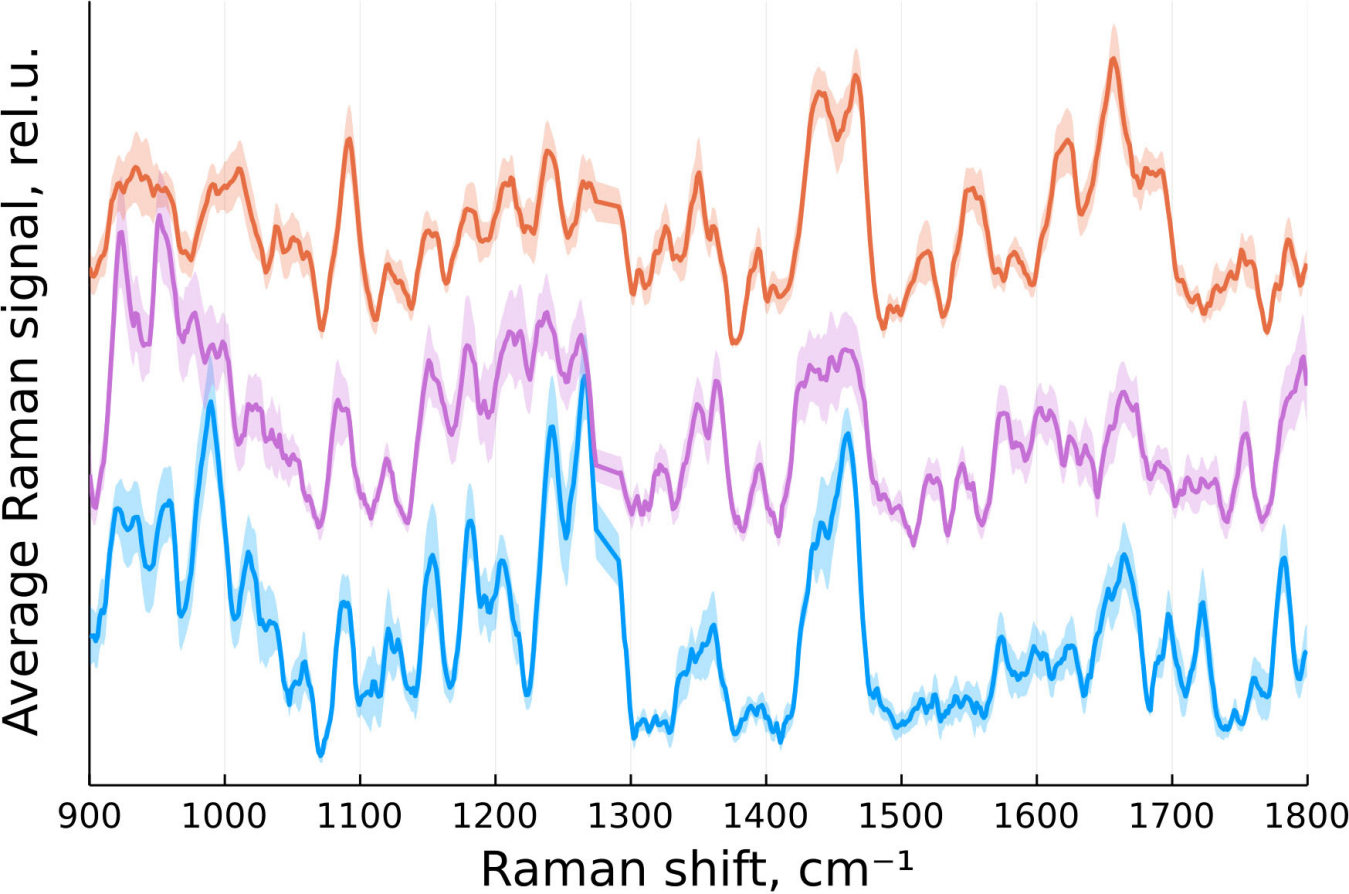
Normalized mean Raman signal from normal brain tissue (blue), tumor edge (magenta) and tumor center (orange) with standard error of the mean indicated by a translucent color.

According to (16), the primary Raman peaks of interest for identifying brain tumors are located between 900 and 1800 cm^-1^. In our case, this range corresponded to 646 data points. The spectra were then normalized by their integral intensity. The averaged spectra for each group of tissue after preprocessing are presented on **Figure 2**.

### 2.3 Dimensionality reduction techniques for Raman spectra

Raman spectra of biological tissues in general and intracranial tumors in particular are characterized by a well-developed structure, which sets researchers the task of reducing the considered characteristic peaks (features) in order to ensure optimal classification of the studied samples by tissue type. As mentioned in the introduction, dimensionality reduction methods fall into two broad classes: feature selection methods and feature projection methods. The feature selection method is designed to find a subset of initial features (peaks, attributes). Methods based on feature projection transform data from high-dimensional space to low-dimensional space, while new features are a combination of a number of old features. In our study, we tried both approaches and also used a combination of them.

#### 2.3.1 The feature selection method and biochemical components

Among the feature selection methods, we chose not wrapping approaches, but filtering methods that use third-party estimates to assess the quality of selection. As such a metric, we chose the result of a test of statistical significance of differences between the studied types of tissues: the norm, the edge of the tumor and the center of the tumor. Based on the findings of the pathologist, our measurements were divided into three classes, including 14 measurements of normal-appearing brain tissue, 15 measurements of the margin of glioblastoma, and 19 measurements of the center of glioblastoma. Then, for each point of the spectrum, the value of the Fisher criterion was calculated. If it exceeded the critical value, we assumed that the given point of the spectrum was significant and left it for further consideration; otherwise, we discarded it from the data vector.

Since one of the important advantages of feature selection methods over feature projection methods is the clarity of their result for the end user, the next step was to search for a match between the remaining features and the known peaks of biochemical components. As such a library of peaks, we used the work (16).

#### 2.3.2 Principal component analysis

This is one of the main ways to reduce the dimensionality of the data, losing the least amount of information. Invented by C. Pearson in 1901 (17) and has since found application in many areas, including chemometrics, bioinformatics, artificial intelligence systems, wherever there is a redundant set of features. The calculation of the principal components consists in calculating the eigenvectors and eigenvalues of the covariance matrix of the initial data, ordering them, and choosing the principal components corresponding to the maximum eigenvalues.

### 2.4 Classification model

We chose the support vector machine (SVM) as the classification method. This method belongs to the family of linear classifiers and can also be considered as a special case of Tikhonov regularization. Each data object is represented as a vector (point) in p-dimensional space. Each of these points belongs to only one of the considered classes. The question is whether the points can be separated by a hyperplane of dimension (p − 1). There can be many such hyperplanes, so it is believed that maximizing the gap between classes contributes to a more confident classification. If such a hyperplane exists, it is called an optimal separating hyperplane, and the corresponding linear classifier is called an optimal separating classifier. The linear classifier does not always give an optimal partition, so it is possible to move to a higher-dimensional space in order to obtain a separating hyperplane of a more complex shape in the original space (18). Such a transition is made with the help of a kernel function, which in our case was radial basis function.

The accuracy, sensitivity and specificity measures were used to evaluate the performance of the SVM classifier.

## 3 Results and discussion

### 3.1 The feature selection and corresponding biochemical components

**Figure 3:**
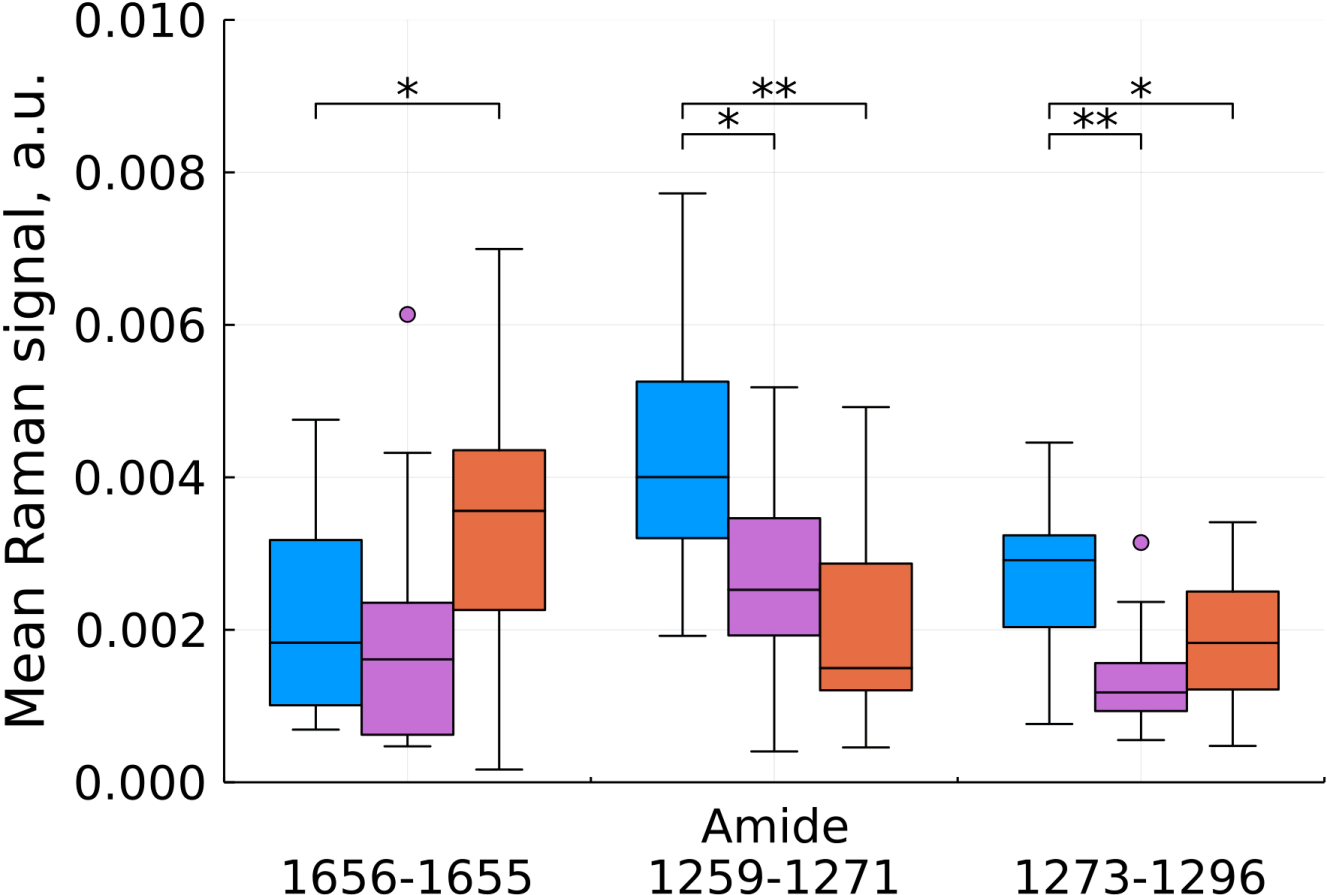
Mean Raman signal in amide bands (blue – normal tissue, magenta - tumor edge, orange - tumor center). Numbers in horizontal labels denote the range of Raman shifts. *p<0.05, **p<0.001.

**Figure 4:**
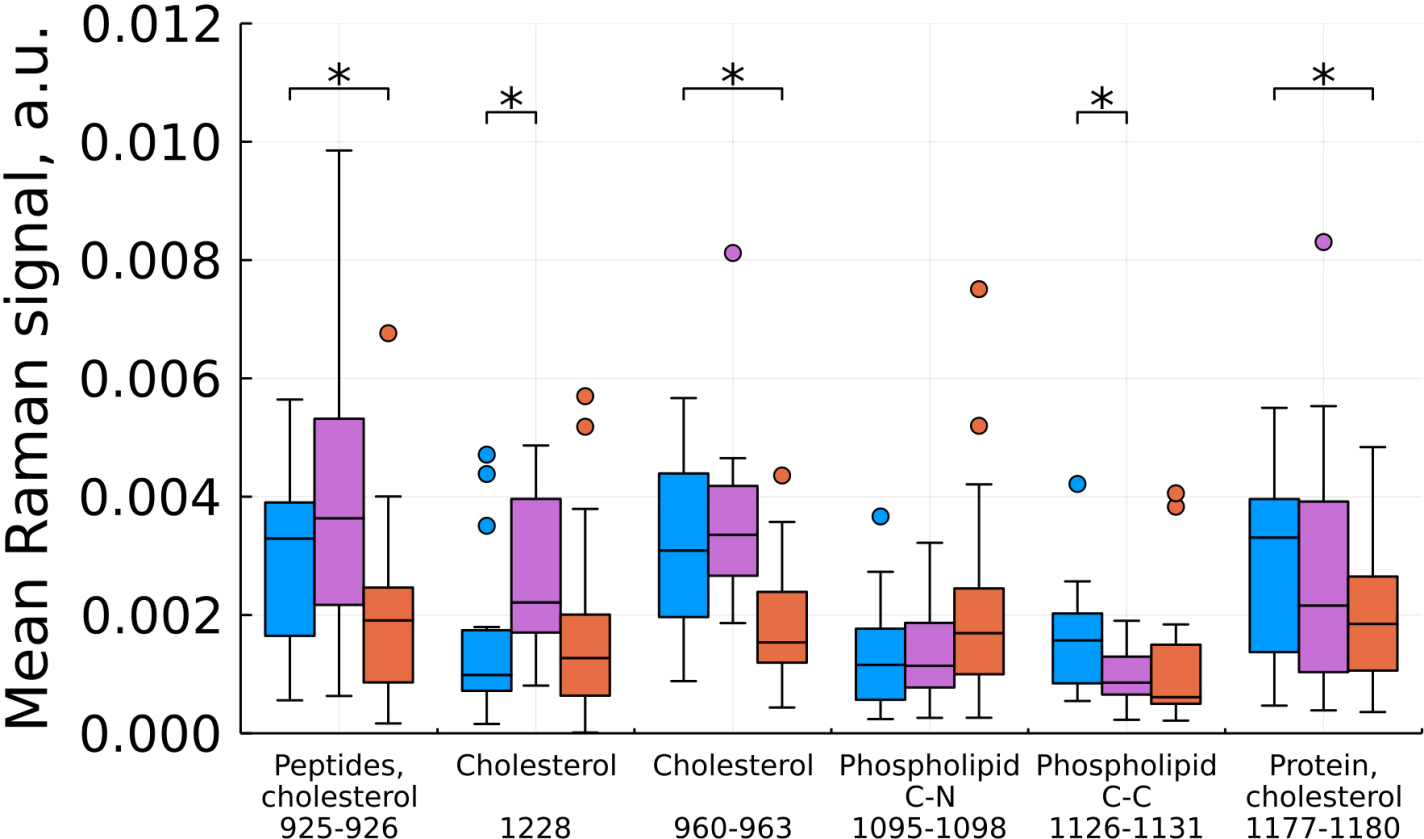
Mean Raman signal in bands of cholesterol, phospholipids and protein (blue – normal tissue, magenta - tumor edge, orange - tumor center). Numbers in horizontal labels denote the range of Raman shifts. *p<0.05.

**Figure 5:**
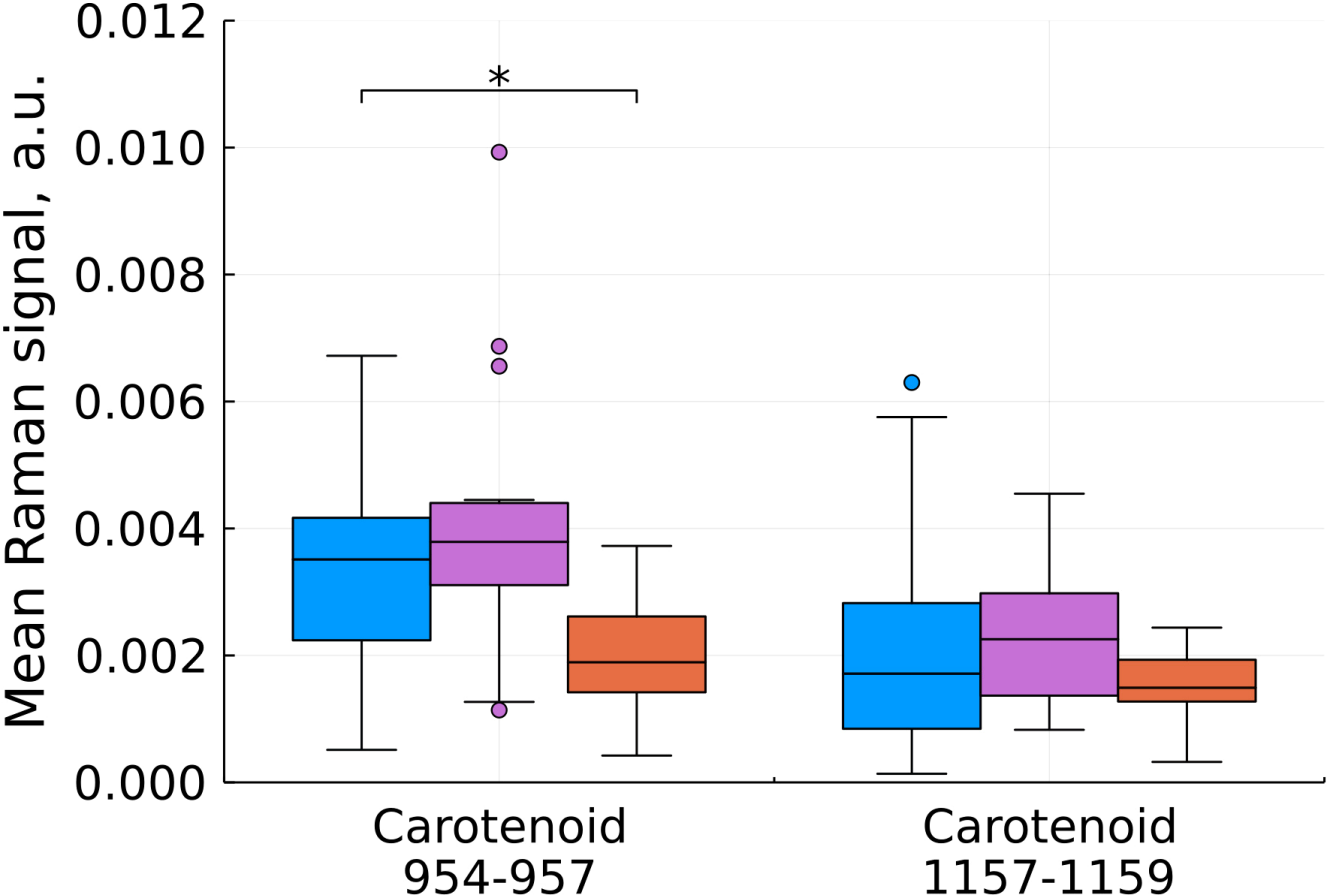
Mean Raman signal in bands of carotenoids (blue – normal tissue, magenta - tumor edge, orange - tumor center). Numbers in horizontal labels denote the range of Raman shifts. *p<0.05.

**Figure 6:**
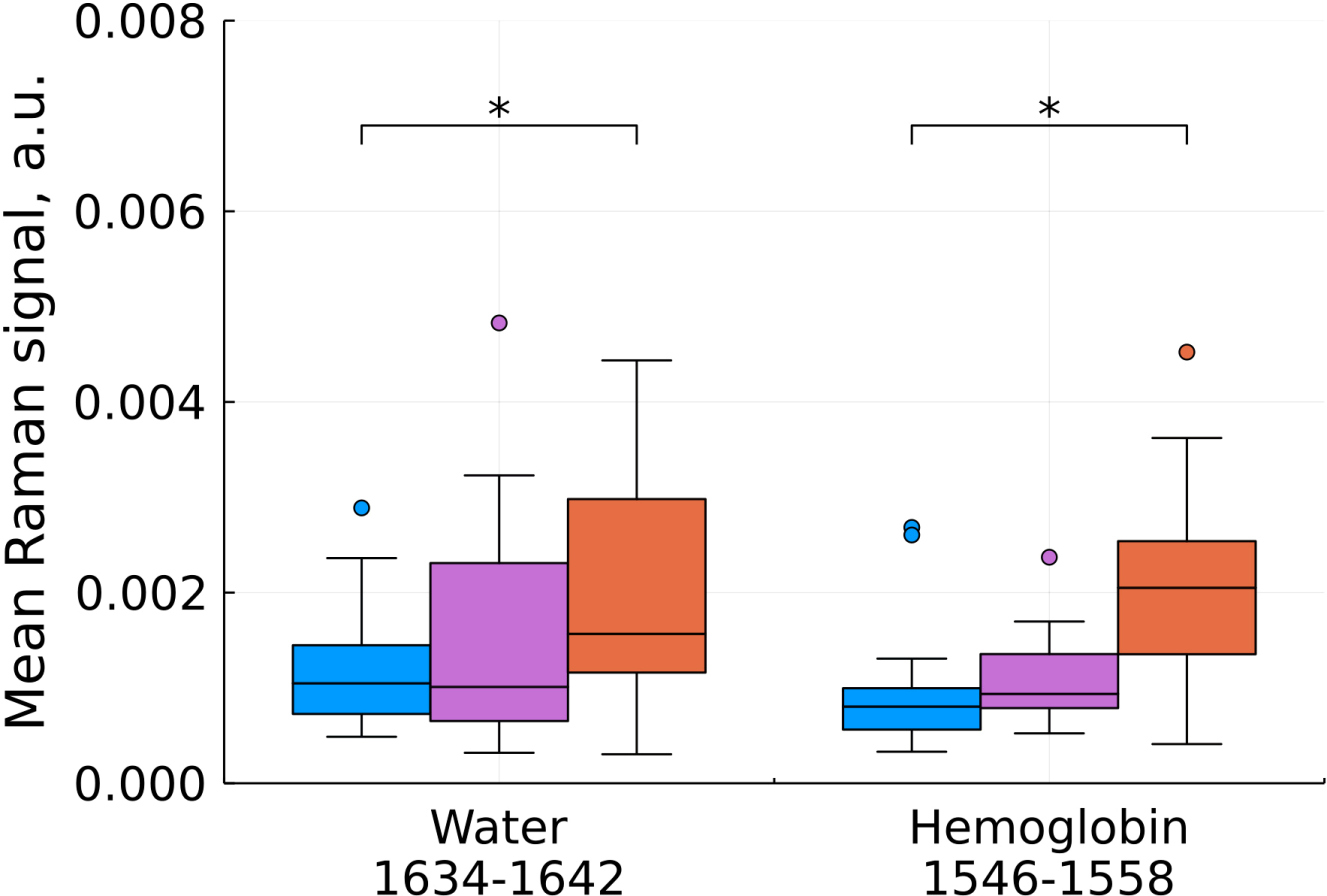
Mean Raman signal in bands of water and hemoglobin (blue – normal tissue, magenta - tumor edge, orange - tumor center). Numbers in horizontal labels denote the range of Raman shifts. *p<0.05.

As a result of feature filtering, statistically significant differences were found between the studied groups of tissues in a number of values of shifts in the Raman spectra. After that, a transition was made to summing the intensities within the remaining spectral ranges, which correspond to important biochemical components of healthy and tumor tissues (**Figures 3**–**6**).

Analysis of changes in the amide bands showed ambiguous results (**Figure 3**), however, a significant contribution of hemoglobin to the 3rd amide band needs to be taken into account and interpreted during further data collection for statistical analysis.

Based on our data, we found a statistically significant decrease in the intensity at shifts corresponding to cholesterol for the tumor compared to the norm and for the tumor compared to the edge, while the edge in some cases (by 960 and 1228 cm^-1^) showed a higher content cholesterol (**Figure 4**). For phospholipids at this stage of the study, there are no unambiguous conclusions. Proteins showed an ambiguous pattern in those peak positions that merge with the peaks of cholesterol (926 and 1178 cm^-1^). At the same time, for the C-N region of the protein (1097 cm^-1^), we see an increase in intensities in the tumor compared to the norm.

Comparison of the characteristic values for carotenoids showed a statistically significant decrease in the intensity during the development of the tumor process in the center of the tumor (**Figure 5**). Carotenoids play an important role in the healthy brain’s antioxidant defense system. The concentration of carotenoids in blood plasma is inversely related to the risk of developing cancer according to epidemiological and experimental studies (19). In the work of (7) a distinct decrease in the intensity of the peaks at 1157 and 1521 cm^-1^ with an increase in the grade of gliomas also was observed.

An increase in hemoglobin content at shifts of 1546-1558 cm^-1^ is in good agreement with the known increase in the blood filling of tumor tissues compared to normal ones (**Figure 6**). One of the most widely used prognostic criteria in determining the degree of malignancy of a tumor is a change in the vascular structure and, as a consequence, the blood filling of the tumor, usually determined by preoperative MRI (20). Blood filling correlates with the level of vascularization and the degree of malignancy of gliomas. H.J. Aaronen et al. showed that the ratio of blood filling of the altered tissue relative to the normal one is 3.64 ± 1.59 for glioblastoma (average ± SD), while for benign tumors this value is close to one (21).

We also found a statistically significant increase in the water content in the samples, judging by the Raman spectra (**Figure 6**) which is consistent with previous studies (22). The increased water content in glial tumors is due to a combination of a number of factors, such as the increased vascularization of tumor tissues, which was mentioned above, the formation of edema, and the presence of body fluids around the necrotic debris that characterize the center of glioblastoma (23).

### 3.2 Principal component analysis

**Figure 7:**
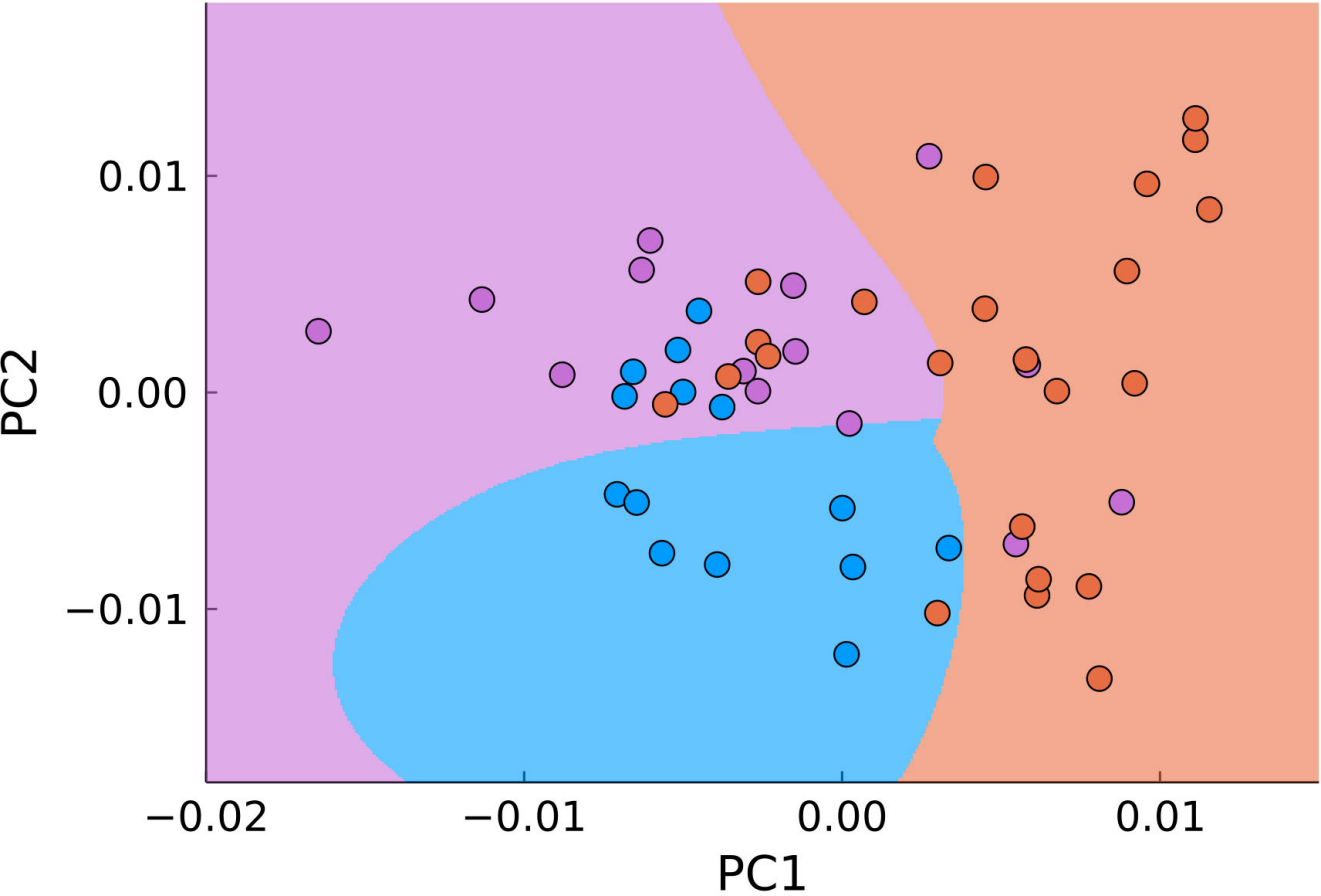
PCA of all points in the range of 900–1800 cm^-1^ (blue – normal tissue, magenta - tumor edge, orange - tumor center). The background color signifies the classification by SVM.

Another approach to dimensionality reduction which is the most widely used in biological applications of Raman spectroscopy is the principal component analysis. This approach seeks to automatically minimize the dimensionality of the measured data. For carrying out the PCA we used MultivariateStats.jl module of Julia programming language. Using PCA allowed reducing the number of dimensions for the Raman shift range of 900-1800 cm^-1^ from 646 to two (**Figure 7**). The data was then used to train Support-Vector Machine classifier (Python 3 library scikit-learn) for automatic detection of malignant samples (**Figure 6**, filled areas), which separated the PC-space into three areas, corresponding to normal, tumor edge and tumor center. We used 30% randomly selected samples to train the classifier with at least 5 samples from each type of tissue. The accuracy of the used classifier for detecting malignant tissue (joint tumor edge and center) was 83% with sensitivity of 96% and specificity of 44%.

### 3.3 SVM classification of pre-filtered features

We applied SVM to the 26 previously filtered features with the same sampling for the training set as in section 3.2. The accuracy of the trained classifier was 83% with 92% sensitivity and 56% specificity.

### 3.4 Combined technique of dimensionality reduction

**Figure 8:**
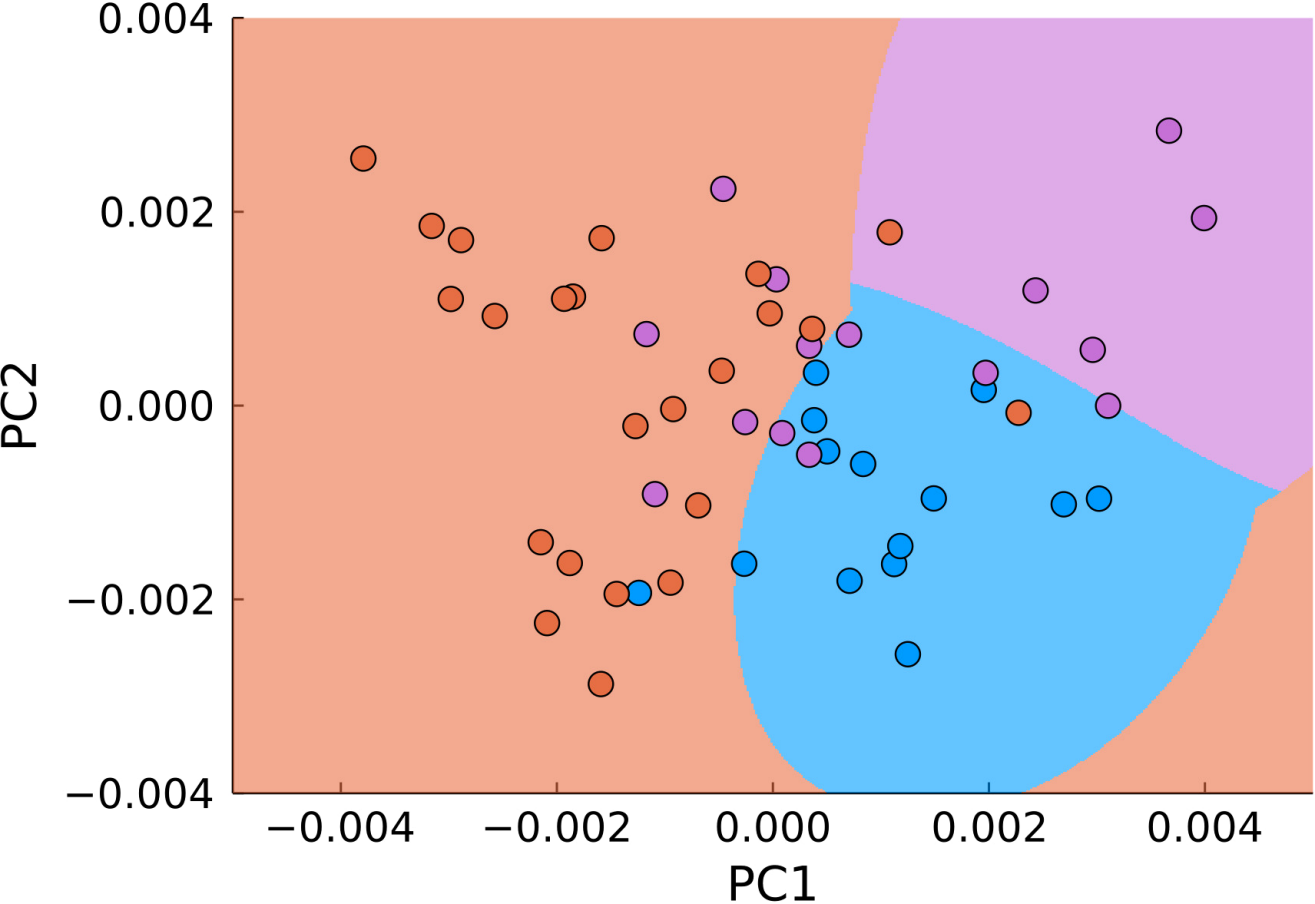
PCA of pre-filtered data (blue – normal tissue, magenta - tumor edge, orange - tumor center). The background color signifies the classification by SVM.

A natural next step in utilizing both approaches is their combination. For that, we applied PCA and SVM not to the entire data range, but to the previously filtered data (**Figure 8**). Using the same parameters for classification, we obtained 83% accuracy with 77% sensitivity and 92% specificity for differentiation of malignant (joint tumor edge and center) and normal tissue.

## 4 Conclusion

In the present work, we have considered two different approaches to dimensionality reduction of the Raman spectroscopy data for analysis of molecular features of intracranial glioblastoma multiforme. As a filtering feature technique, we used the selection of spectral points that showed statistically significant differences between the investigated groups of tissues, followed by comparison of them and the spectral ranges of known biochemical components. As a feature projection method, we used the principal component analysis. The support vector machine was used as a classification algorithm. The approach that combines preliminary feature filtering with PCA provided significantly higher specificity at the cost of reduced sensitivity compared to both the approach using only PCA over spectral data and the approach of the classification algorithm for biochemical components without PCA. The accuracy of each method remained the same. It should be noted that to better train the classifier, the measurements from the same patient should not be sampled into both the training and the testing sets. The obtained result allows us to conclude about the advantages of the two-stage dimensionality reduction algorithm both in terms of improving the quality of classification and in terms of a more visually convenient representation of the detected differences between classes due to the preliminary selection of biochemical components. In this paper, we propose a basic approach to data analysis, but further expansion of the sample is required to build an automatic classification system that works in the clinic. We also plan to expand the set of optical-spectral features using fluorescence and diffuse reflectance spectroscopy.

## Data availability statement

The raw data supporting the conclusions of this article will be made available by the authors, without undue reservation.

## Ethics statement

This study was reviewed and approved by N.N. Burdenko National Scientific and Practical Center for Neurosurgery ethics committee. The patients/participants provided their written informed consent to participate in this study.

## Author contributions

Spectroscopic measurements, spectrum pre-processing, principal component analysis, classification algorithm development—IR. Analysis of biochemical components—IR, TS. Filtering approach to dimensionality reduction of Raman spectra—TS, AO. Collection and preliminary analysis of clinical material—AKo, VO, SG. Histopathological analysis of collected tissue samples—SS. Clinical design of the experiment—AKr, SG, IP. Biological design of the experiment—DG, GP. Design of the experiment—IR, TS, VL. Manuscript editing and revision—IR, TS. All authors contributed to the article and approved the submitted version. 

## Funding

This work was supported by a Ministry of Science and Higher Education of the Russian Federation (agreement 075-15-2021-1343, October 4, 2021).

## Conflict of interest

The authors declare that the research was conducted in the absence of any commercial or financial relationships that could be construed as a potential conflict of interest.

## Publisher’s note

All claims expressed in this article are solely those of the authors and do not necessarily represent those of their affiliated organizations, or those of the publisher, the editors and the reviewers. Any product that may be evaluated in this article, or claim that may be made by its manufacturer, is not guaranteed or endorsed by the publisher.
